# Investigating impacts of small dams and dam removal on dissolved oxygen in streams

**DOI:** 10.1371/journal.pone.0277647

**Published:** 2022-11-17

**Authors:** Katherine M. Abbott, Peter A. Zaidel, Allison H. Roy, Kristopher M. Houle, Keith H. Nislow

**Affiliations:** 1 Massachusetts Cooperative Fish and Wildlife Research Unit, University of Massachusetts Amherst, Amherst, Massachusetts, United States of America; 2 U.S. Geological Survey, Massachusetts Cooperative Fish and Wildlife Research Unit, University of Massachusetts Amherst, Amherst, Massachusetts, United States of America; 3 Division of Ecological Restoration, Massachusetts Department of Fish and Game, Boston, Massachusetts, United States of America; 4 Northern Research Station, U.S.D.A. Forest Service, University of Massachusetts Amherst, Amherst, Massachusetts, United States of America; Penn State University, UNITED STATES

## Abstract

Small surface-release dams are prevalent across North American watersheds and can alter stream flow, thermal regimes, nutrient dynamics, and sediment transport. These dams are often implicated as a cause of negative water quality impacts—including reduced dissolved oxygen (DO)—and dam removal is increasingly employed to restore natural stream processes and improve DO. Published impacts of small dams on DO vary widely across sites, and even less is known about the extent and timescale of DO recovery following removal. Therefore, we sought to quantify the effects of small dams and dam removal on DO and determine the dam, stream, and watershed characteristics driving inter-site variation in responses. We deployed continuous data loggers for 3 weeks during summer months in upstream (reference), impoundment, and downstream reaches at each of 15 dammed sites and collected equivalent data at 10 of those sites following dam removal. Prior to dam removal, most sites (60%) experienced a decrease in DO (an average of 1.15 mg/L lower) within the impoundment relative to upstream, but no consistent impacts on diel ranges or on downstream reaches. Before dam removal, 5 impacted stream reaches experienced minimum DO levels below acceptable water quality standards (<5 mg/L); after dam removal, 4 of 5 of these reaches met DO standards. Sites with wider impoundments relative to upstream widths and sites located in watersheds with more cultivated land experienced the greatest decreases in impoundment DO relative to upstream. Within one year following dam removal, impoundment DO recovered to upstream reference conditions at 80% of sites, with the magnitude of recovery strongly related to the magnitude of pre-removal impacts. These data suggest that broadly, small dams negatively affect stream DO, and the extent of effects are modulated by impoundment geometry and watershed characteristics. These results may help practitioners to prioritize restoration efforts at those sites where small dams are having outsized impacts, and therefore where the greatest water quality benefits may occur.

## Introduction

Dams are pervasive features of global watersheds [1, 2] and have the potential to significantly impact stream ecology by altering water quality and hydrology, impeding sediment and nutrient transport, and fragmenting biotic assemblages [3]. Small, surface-release dams (<15 m high) constitute the majority of dams in the U.S., and, despite their relatively small stature, have been shown to negatively impact water quality both within impoundments [4, 5] and downstream [6–8]. In contrast to larger water storage dams, small surface-release dams often operate as run-of-river projects and are less likely to modify downstream flows [3]. With over 2 million dams in the United States [9], the water quality impacts of even small dams may be magnified across the landscape, carrying significant implications for stream ecosystems and their biota [10]. With the goals of mitigating negative ecological impacts, and for public safety and cost considerations, small dam removal has become an increasingly prevalent stream restoration method both within the U.S. [11] and internationally [12, 13]. Despite the increasing number of dam removals, few streams have been consistently monitored before and after dam removal, and few studies document the extent of water quality impacts across a range of dam, stream, and watershed characteristics [14].

Dissolved oxygen (DO) is one such critical water quality parameter that may be impacted by dams and dam removal. This chemical property of water shapes biogeochemical processes, stream metabolism, and biotic assemblages [15, 16], and is affected by both physical (e.g., temperature and turbulence) and biological (e.g., photosynthesis and respiration) mechanisms [17]. Alterations to these mechanisms may change DO concentrations and, subsequently, how a stream processes and transports organic matter and nutrients [17, 18]. Small dams may reduce atmospheric mixing and turbulence by slowing impoundment flow velocities [19] and can increase primary production and decomposition by facilitating the accumulation of sediment and promoting warmer waters [4, 6, 20]. Decreases in impoundment DO levels, coupled with warmer temperatures, may limit the abundance of sensitive taxa through metabolic stress and the interactive and additive stressors of increased nutrient fluxes and contaminants—such as methyl mercury—released in anoxic conditions [21, 22]. Low DO impoundment waters could result in decreased downstream DO concentrations, although reaeration and oxygen entrainment from surface waters spilling over dams may negate this effect. By returning an impounded stream to a lotic state, dam removal has the potential to increase flow velocity and surface water mixing, reduce residence time, and reduce radiant heating as canopy cover develops [23]. These changes may lead to DO regimes downstream and within the former impoundment that are more consistent with upstream, unimpacted reaches.

Despite the importance of DO to stream ecological processes, research focusing on the impacts of dams and dam removal on DO has typically been limited to discrete or short-term (e.g., <48 hours) measurements or small-scale (e.g., <3 sites) studies [21, 24–26], and studies specifically examining DO impacts of small dams have found mixed results. Unlike bottom-release dams that consistently release low DO hypolimnetic water to downstream reaches [27, 28], the downstream DO impacts of surface-release dams exhibit considerable variation among sites, with increased [29], unchanged [4, 30], and reduced [6] DO concentrations observed downstream. Impacts to the impoundment are more consistently negative, with decreased mean DO [6, 29] and larger diel ranges [5, 6] relative to upstream lotic reaches. This variability in water quality across dammed sites [31] suggests water quality recovery following dam removal may also vary across sites. Although the general mechanisms by which dam removal may impact water quality are known, documented responses to dam removal vary widely across studies. For example, August impoundment DO concentrations in the Middle Cuyahoga River (Ohio, U.S.) averaged below 5 mg/L, and following partial removal of the Kent Dam, DO increased to between 6 and 9 mg/L [21]. Other studies, however, found relatively minimal changes in DO concentrations following dam removal [24, 25].

Given that each dam and dam removal is unique and nested within a distinct stream system and watershed, a case study approach (i.e., focusing on one or few dam sites) may not be sufficient or appropriate to understand broader water quality impacts and restoration responses across a state or region. In this study, we therefore examined stream DO concentrations before and after dam removal at sites representing a range of dam, stream, and watershed characteristics found in the Northeastern U.S. We first quantified the effects of small dams on impoundment and downstream DO concentrations relative to upstream reference reaches, and determined the characteristics influencing inter-site variability in dam impacts. We then quantified the responses of impoundment and downstream DO concentrations to dam removal and determined the site- and watershed-specific characteristics influencing responses to dam removal. The results of this study provide a more comprehensive understanding of not only the ecological consequences of small dams, but also the recovery potential following dam removal. Incorporating a range of environmental predictors may also support better predictions of where the most negative DO impacts may be found across a dammed landscape, and which rivers could benefit the most from dam removal.

## Methods

### Study area

**Fig 1:**
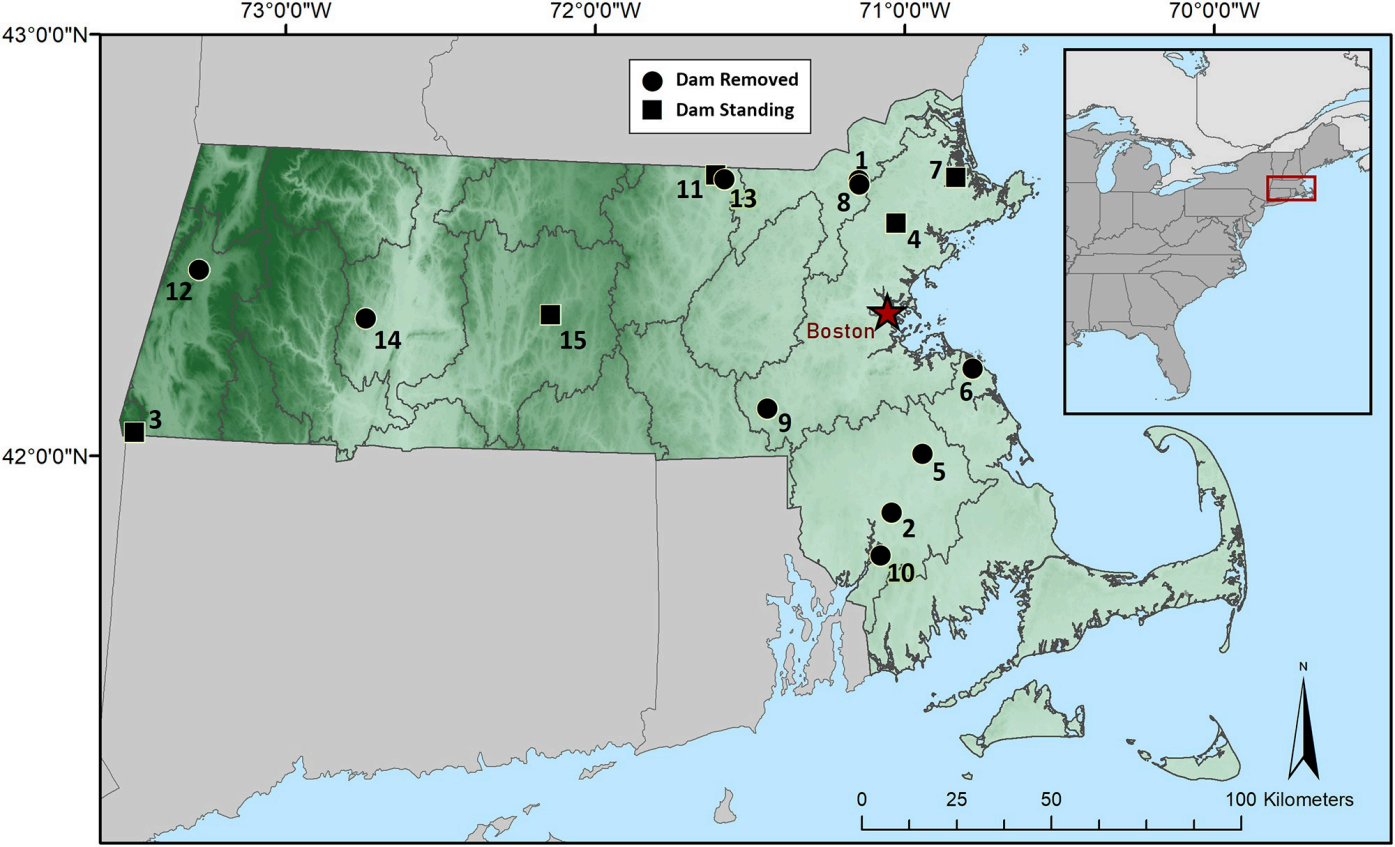
Study sites. Map of the 15 dams in Massachusetts, USA, monitored within this study, of which 10 (circles) were removed over the course of this study and 5 (squares) remained standing. See Table 1 for site information corresponding to numbers. Map was prepared using ArcGIS 10.8 (Esri, Redlands, California, USA).

We assessed the impacts of small dams and dam removal on DO at 15 surface-release dam sites in Massachusetts, USA (Fig 1, Table 1). Data collection for this study was conducted in streams with recent (2015–2020; n = 10) or planned (n = 5) dam removals. As the third most densely populated state in the U.S. [32], Massachusetts contains large cities (i.e., Boston, Worcester, Springfield), sprawling suburbs, rural towns, and agricultural land, with concomitant ranges in impervious cover, pollution, and habitat alteration. Sites were selected to capture a range of dam, stream, and landscape characteristics, including watershed size (3–389 km^2^), forest cover (25–90%), cultivated land cover (0–2.6%), and impervious cover (0–27%; Table 2). One site (site 14) required an access permit which was approved by the City of Northampton Department of Public Works. No other sites required permits for access, and permissions from dam and landowners were obtained.

**Table 1:** Site information for the 15 dams throughout Massachusetts, USA, monitored within this study.

Site	Site Name	Stream Name	Removal Year	Dam Height (m)	Impoundment Area (ha)	Drainage Area (km^2^)	Forest Cover (%)
1	Balmoral	Shawsheen River	2017	2.1	2.3	188.8	25.3
2	Barstow’s Pond	Cotley River	2018	2.6	4.1	19.4	66.6
3	Becker’s Pond	Unnamed tributary to Schenob Brook	-	4.3	0.3	2.7	80.8
4	Bostik / S. Middleton	Ipswich River	-	3.1	7.5	113.4	31.7
5	Cotton Gin Mill	Satucket River	2017	1.5	0.1	55.4	41.2
6	Hunter’s Pond	Bound Brook	2017	3.4	0.5	29.5	72.5
7	Ipswich Mills	Ipswich River	-	3.2	13.1	388.5	49.5
8	Marland Place	Shawsheen River	2017	3.8	2.1	183.9	25.5
9	Old Mill	Charles River	2017	4.1	3.1	65.5	46.3
10	Rattlesnake Brook	Rattlesnake Brook	2016	1.2	1.5	17.1	89.7
11	Sucker Brook	Sucker Brook	-	1.2	1.3	6.8	50.1
12	Tel-Electric	West Branch Housatonic River	2020	6.1	4.4	93.5	59.4
13	Millie Turner	Nissitissit River	2015	3.1	6.9	155.1	78.7
14	Upper Roberts Meadow	Roberts Meadow Brook	2018	10.7	1.7	22.8	87.3
15	Wheelwright	Ware River	-	5.1	21.4	334.1	74.3

Note: Dash symbols (-) indicate that no dam removal occurred over the course of this study.

**Table 2:** Dam, stream, and watershed characteristics for the 15 sites monitored within this study.

Characteristic	Minimum	Average	Maximum
Dam height (m)	1.2	3.7	10.7
Impoundment surface area (ha)	0.1	4.7	21.4
Impoundment volume (m^3^)	200	72383	308370
Impoundment widening* (ratio)	1.2	7.9	57.4
Impoundment length:width ratio	0.7	43.2	146.3
Impoundment residence time (hrs)	0.4	771.0	8916.1
Upstream slope (%)	0.0	0.3	1.2
Downstream slope (%)	0.0	0.5	1.5
Watershed area (km^2^)	2.7	111.8	388.5
Forest cover (%)	25.3	58.6	89.7
Impervious cover (%)	0.1	10.2	27.6
Cultivated cover (%)	0.0	0.5	2.6
Wetland cover (%)	4.3	12.4	21.8

* impoundment width:upstream reference width

### Dissolved oxygen data collection

To assess the impacts of dams and dam removal on DO, Onset© U26 Dissolved Oxygen data loggers (U26-001; Onset Computer Corporation, Bourne, MA, USA) were deployed for week-long periods during each of July, August, and September, and recorded DO concentrations every 15 min. Warm, summer months were targeted for data collection when water levels were expected to be low, temperatures were highest [8], and mean DO would likely be lowest; thus, our results likely represent the worst-case effect of dams on DO. The typical sampling duration across sites was 3 deployments per year for 1–2 years before removal and 1–2 years after removal. The exact number of deployments and the years loggers were deployed varied widely by site (pre-removal range: 3–16, post-removal range: 3–9) due to the timing of dam removals (Table A in S1 Appendix; Table 1). Concurrent temperature data were also collected. At each of the 15 dam sites, data loggers were deployed within 3 reaches: *upstream* of the maximum extent of the impoundment, within the *impoundment* floating just below the surface, and *downstream* of the dam. *Impoundment* refers to the lentic waterbody artificially created by the dam and the extent of the impoundment was determined by Google Earth Pro (version 7.3.4.8248) and field observations. The downstream loggers were installed a minimum of 20 m from the dam to minimize immediate effects of spillway reoxygenation (distance range; 20–65 m downstream). After dam removal (10 sites), data loggers were deployed in the same upstream and downstream reaches, and within the former impoundment.

In the field, we used a multiparameter probe (HI 9829, Hanna Instruments Inc., Woonsocket, RI) to determine logger locations that were representative of DO concentrations for each reach and to collect field data points with which to correct loggers for drift. Loggers were deployed in white polyvinyl chloride (PVC) flow-through housings to physically protect the loggers and shield them from direct solar radiation [33, 34]. Loggers deployed in flowing-water reaches were anchored on rebar (~10–20 cm above the stream bottom), while impoundment loggers were hung from anchored floats ~30 cm below the water surface. Impoundment loggers were deployed ~25 m upstream of the dam for safety reasons. At each impoundment logger, we also conducted vertical DO and temperature profiles with a multiparameter probe at 0.5-m intervals from the surface down to ~0.5 m above the impoundment bottom.

In the laboratory, DO loggers were calibrated to 100% oxygen saturation using a wetted sponge enclosure and to 0% oxygen saturation using a sodium sulfide solution prior to each week-long deployment. Following retrieval and data downloads, dissolved oxygen data were quality controlled (QC) to remove data points measured on deployment and retrieval days (i.e., incomplete records). Data were plotted and visually inspected, and quantitatively examined using the *ContDataQC* package [35] in R [36]. Anomalous data points (i.e., extreme gross values, rates of change, or flat values) taken when the logger may have been fouled or out of water were removed from analyses. Logger values were compared to those from a multiparameter probe at retrieval from the field to ensure biofouling was not affecting the data recording, and data were corrected for drift using HOBOware^®^ Pro Dissolved Oxygen Assistant.

### Dam, stream, and watershed variables

Dam-specific characteristics (i.e., heights and impoundment volumes) were obtained via engineering technical reports provided by Massachusetts Division of Ecological Restoration or through the National Inventory of Dams (U.S. Army Corps of Engineers; http://nid.usace.army.mil/). Where impoundment volumes were not available, volumes were estimated via the average end area method [37], in which the cross-sectional area of the impoundment was estimated for the upstream- and downstream-most extents and averaged across the length of the impoundment. Impoundment surface area was calculated using the Massachusetts Department of Environmental Protection (MassDEP) Hydrography (1:25,000) data layer (Massachusetts Bureau of Geographic Information (MassGIS)). To represent the geomorphic impact of the dam, impoundment widening was calculated in Google Earth Pro (version 7.3.4.8248) by averaging three evenly spaced width measurements over the length of the impoundment and dividing by the average upstream width determined from field measurements [8]. Impoundment length:width ratio was calculated by dividing the flowing length of the impoundment by the average width of the impoundment as measured in Google Earth Pro.

We estimated reach-scale stream gradient (percent slope) by averaging 4 adjoining 30 m-cells around each logger location using available data from the Designing Sustainable Landscapes: Stream gradient settings variable, which is derived from National Hydrography Dataset 1:25,000 flowlines and a depression-less version of the National Elevation’s Dataset 10 m Digital Elevation Model [38]. Daily discharge data for each dam site were estimated from U.S. Geological Survey (USGS) gages, which were selected for geographic proximity and similar watershed size to each study stream (Table B in S1 Appendix). We used the drainage-area ratio method [39] to estimate daily discharge based on a ratio of gaged:ungaged watershed sizes [40]. We used StreamStats (USGS; http://streamstats.usgs.gov) to delineate the watershed upstream of each dam and to generate watershed area and land cover metrics (e.g., percent of forest, wetland, or impervious cover within a watershed). The percentage of cultivated land within a watershed was calculated using ArcGIS 10.8 tools (Esri, Redlands, CA, USA) using the MassGIS Data: 2016 Land Cover/Land Use dataset (MassGIS; https://www.mass.gov/info-details/massgis-data-2016-land-coverland-use). Where a watershed extended outside of state boundaries (i.e., Site 13), the remaining area was calculated from the National Land Cover Dataset (2016).

### Data analyses

We calculated daily DO metrics (minimum, average, maximum, and diel range) for each reach at each site over each deployment period [41]. To minimize temporal autocorrelation and associated inflated significance values, we subset the data by selecting the first and last days from each deployment period at each site, resulting in 2 daily measurements per deployment period (e.g., 4 pre-removal deployments: n = 8). To quantify dam impacts on impoundment DO, *impoundment effect* was calculated for each metric as the daily difference between the impoundment and upstream mean concentrations at each site, where negative values indicate a lower DO within impoundments relative to upstream. Similarly, we quantified dam impacts on downstream DO by calculating the *downstream effect* as the daily difference between the downstream and upstream values at each site. To assess the response of DO to dam removal, we first summarized DO metrics by month to make comparisons before and after dam removal. We then calculated the *impoundment removal response* as the difference between post- and pre-removal impoundment effects, whereby positive values indicate an increase in post-removal impoundment DO concentrations, relative to upstream. We similarly calculated the *downstream removal response* as the change between pre- and post-removal downstream effects. We focus on raw differences (e.g., impoundment-upstream values), because they are more readily interpreted and retain more information about the context of the data [42]. However, we note that statistical significance may vary due to unavoidable differences in sample sizes among sites; therefore, we also used the *effsize* package in R [43] to calculate the effect size metric Cliff’s delta *d*, which expresses the probability that a randomly selected observation from one reach is larger than a randomly selected observation from another reach [44], and can be generally interpreted as negligible (|<0.147|), small (|<0.33|), medium (|<0.474|) and large (|0.474–1.000|) effects [43].

For statistical tests of DO differences between reaches and before and after dam removal, we performed Wilcoxon Signed Rank (matched pairs) tests following tests of normality (Shapiro-Wilk) using an α level of 0.05 and controlling the false discovery rate using the Benjamini-Hochberg adjustment. To develop models to predict DO effects and responses both pre- and post-dam removal, we built candidate sets of linear mixed effects models (using the *lme4* package in R [45]) for each response variable (e.g., impoundment effect, impoundment removal response) that included additive combinations of dam, stream, and watershed variables as predictors. Site and year were included as random effect terms to focus on inter-site variation in response [45]. Additionally, although this study does not focus on flow impacts on DO, we tested models that included watershed area-normalized daily discharge (log-transformed) as a fixed covariate to account for the effects of daily streamflow on water quality around run-of-river dams [8, 24]. Further, to understand the relationship between impoundment temperature impacts and impoundment DO effects, we tested a single parameter model including impoundment warming (mean impoundment temperature minus mean upstream temperature), with random effects of site and year and discharge as a fixed covariate.

All continuous predictor variables were tested for normality and transformed as necessary. Continuous predictor variables were also Z-score standardized to eliminate differences in the scales of units amongst predictor variables. Pairwise correlations (Table C in S1 Appendix) and variance inflation factors (VIF) identified collinearity amongst predictor variables, and we did not include predictors with Pearson correlation coefficients >0.7 and VIF values >3.0 in further additive models. We did not test more than three predictor variables (not including the random effects) for each response variable to avoid overfitting. Akaike Information Criterion corrected for small sample size (AICc) was used to determine the best supported models [46]. Site 10 was not included in modeling of inter-site variation because the impoundment had two inflowing tributaries. We used R version 4.0.2 to perform all statistical analyses [36].

## Results

### Effects of small dams on stream DO

**Fig 2:**
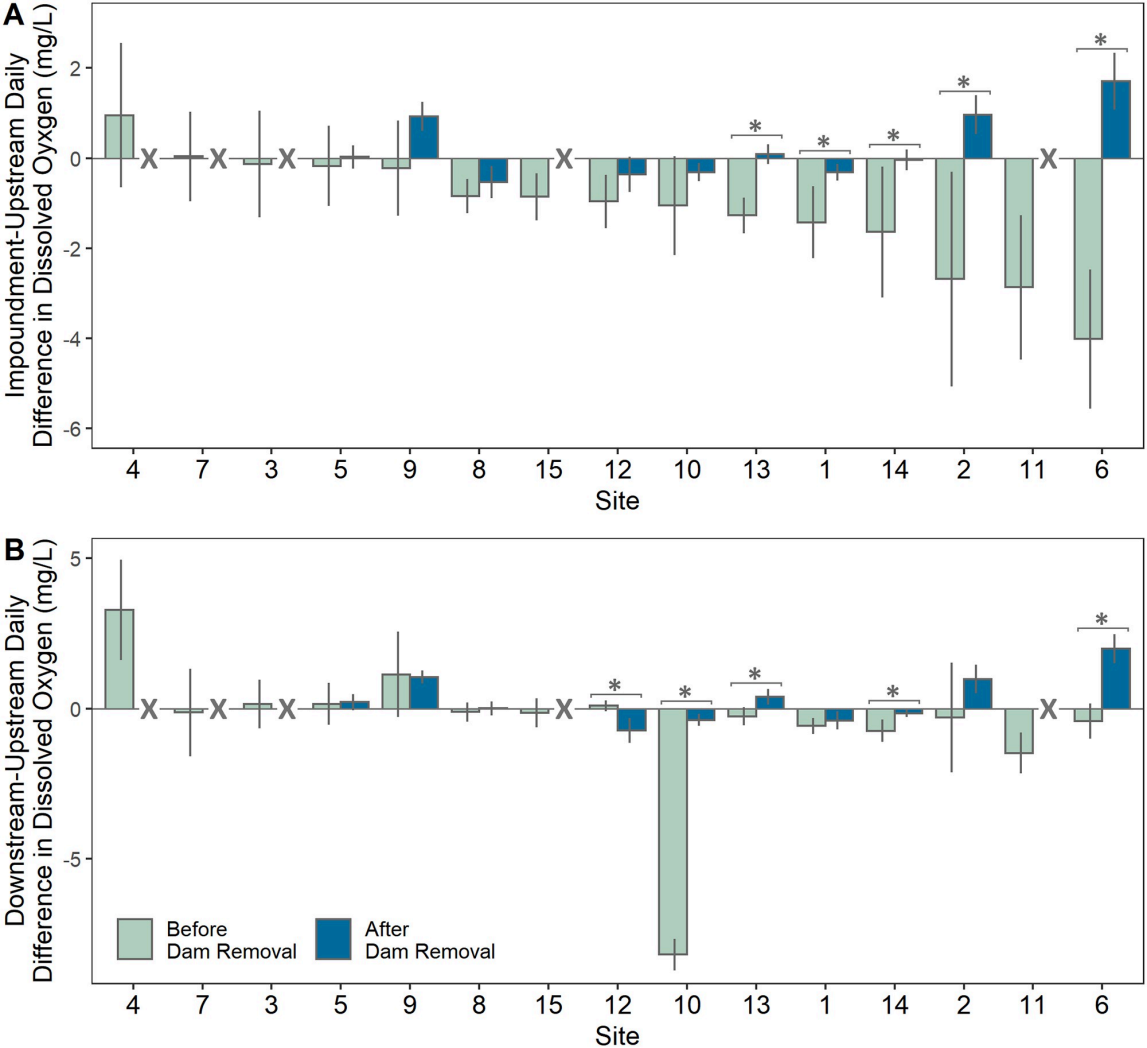
Dissolved oxygen (DO) effects before and after dam removal. (A) Variation in impoundment effect (mean daily impoundment minus upstream DO) by site before and after dam removal. (B) Variation in downstream effect (mean daily downstream minus upstream DO). Error bars represent one standard deviation about the mean. The horizontal line at zero indicates no difference between impoundment or downstream DO and the upstream reference. Significant differences (α = 0.05) between DO effects before and after dam removal are annotated with an asterisk (*) and sites where the dam was not removed during this study are annotated with an X. Sites are ordered from lowest to highest pre-removal impoundment effect.

Most impoundments (13 of 15 sites) exhibited negative DO effects (lower mean daily DO concentrations compared to upstream reference reaches), with 9 impoundments experiencing significantly lower DO than upstream, and 10 experiencing large negative effects as determined by Cliff’s delta (Fig 2A, Table 3). Across the 15 sites, impoundment DO concentrations were an average of 1.15 mg/L lower (range = -4.02 to +0.95 mg/L) than upstream reference reaches. Four impoundments experienced an average minimum surface DO concentration less than 5.0 mg/L which is the threshold used by the Massachusetts Department of Environmental Protection (MDEP) as a concentration below which waters may be considered impaired for DO (MDEP 2016; Table 4). Some impoundments experienced critically low daily oxygen levels, with 13 of 15 impoundments experiencing at least one day of minimum DO less than 5 mg/L, and 5 impoundments experiencing DO concentrations less than 3 mg/L. Despite periodically low DO concentrations, only 8 impoundment or downstream reaches were below the 5 m/L threshold for acceptable water quality standards (Table 4). Discrete vertical profiles taken within 13 impoundments (profiles not taken at 2 sites due to consistently shallow depths) revealed no hypoxia throughout the water column (DO concentrations < 3.0 mg/L), with the exception of site 6, which experienced hypoxic conditions throughout the shallow water column (Table D in S1 Appendix). Oxygen and thermal stratification were not observed at most sites, and no impoundments remained stratified throughout the summer. Before dam removal, impoundments were on average 1.24 degrees (°C) warmer than upstream reaches (range: -0.12 to 4.30 °C), and downstream reaches were on average 0.80 °C warmer than upstream reaches (-0.16 to 4.21 °C; S1 Fig). Five sites experienced impoundment diel ranges over 1.9 mg/L larger than upstream ranges (Table 5).

**Table 3:** Differences in mean daily dissolved oxygen between impoundment and upstream references before and after dam removal, also termed the impoundment DO effect.

Site	Impoundment DO Effect Before Removal					Impoundment DO Effect After Removal				
	DO Effect (mg/L)	*P*	Cliff’s delta *d*	Effect Size (direction)	n	DO Effect (mg/L)	*P*	Cliff’s delta *d*	Effect Size (direction)	n
1	**-1.43**	0.013	-0.84	large (-)	8	-0.32	0.071	-0.39	medium (-)	6
2	**-2.69**	0.012	-0.79	large (-)	14	**0.96**	0.002	0.83	large (+)	12
3	-0.14	0.848	-0.04	negligible (-)	24	-	-	-	-	-
4	**0.95**	0.011	0.34	medium (+)	32	-	-	-	-	-
5	-0.17	0.856	-0.03	small (-)	8	0.02	0.875	0.08	negligible (+)	6
6	**-4.02**	0.013	-1.00	large (-)	8	1.71	0.071	0.89	large (+)	6
7	0.04	0.933	0.23	small (+)	8	-	-	-	-	-
8	**-0.85**	0.013	-0.75	large (-)	8	-0.53	0.179	-0.58	large (-)	6
9	-0.23	0.904	-0.19	small (-)	8	0.93	0.071	0.67	large (+)	6
10	**-1.06**	0.047	-0.81	large (-)	8	**-0.31**	0.002	-0.39	medium (-)	18
11	**-2.87**	<0.001	-0.86	large (-)	24	-	-	-	-	-
12	**-0.96**	0.005	-0.90	large (-)	14	-0.37	0.156	-0.44	medium (-)	6
13	-1.27	0.170	-0.54	large (-)	6	0.09	0.625	0.06	negligible (+)	6
14	**-1.64**	0.012	-0.71	large (-)	14	-0.04	0.625	-0.03	negligible (-)	8
15	**-0.86**	0.002	-0.66	large (-)	18	-	-	-	-	-

Notes: Bold font indicates a significant difference between impoundment and upstream mean DO (e.g., impoundment-upstream) at α = 0.05 as determined by Wilcoxon Rank-Sign tests. Column heading “n” indicates number of daily measurements included in analyses (e.g., first/last day of each logger deployment). Dash symbols (-) indicate that no dam removal occurred over the course of this study. Negative values indicate impoundment DO is lower than upstream DO. Cliff’s *d* can be interpreted as negligible (|<0.147|), small (|<0.33|), medium (|<0.474|) and large (|0.474–1.000|) effects.

**Table 4:** Average daily minimum dissolved oxygen (DO) concentration in upstream (US), impoundment (IMP), and downstream (DS) reaches before and after dam removal. Overall minimum (i.e., lowest concentration recorded) in parentheses. Blue shading represents average minimum DO concentrations >6 mg/L, green shading represents <6 and >5 mg/L, and orange shading represents <5 mg/L, a threshold below which waters are considered impaired for DO (MDEP 2016). Grey shading and dash symbols (-) indicate no dam removal occurred over the course of this study.

Site	Average Minimum DO (mg/L) Before Dam Removal			Average Minimum DO (mg/L) After Dam Removal		
	US	IMP	DS	US	IMP	DS
1	7.48 (6.54) (6,54)	5.75 (4.81)	6.93 (5.93)	7.91 (7.07)	7.39 (6.40)	7.31 (5.92)
2	5.51 (3.54)	1.12 (0.00)	4.27 (2.81)	6.40 (4.66)	6.80 (6.10)	6.91 (5.84)
*3	7.29 (5.71)	7.04 (4.28)	7.44 (3.37)	-	-	-
4	2.60 (0.00)	3.43 (0.62)	6.11 (0.26)	-	-	-
5	5.50 (2.86)	5.28 (3.58)	5.92 (4.26)	5.33 (4.47)	5.58 (5.18)	5.55 (4.92)
6	4.13 (1.40)	0.51 (0.00)	4.14 (1.28)	4.96 (4.30)	4.50 (2.52)	5.43 (4.24)
7	5.24 (4.79)	6.00 (4.75)	2.03 (0.87)	-	-	-
8	7.03 (6.45)	5.95 (5.01)	7.60 (6.55)	7.64 (6.87)	7.17 (6.69)	7.80 (6.97)
9	6.26 (3.60)	5.95 (4.54)	7.71 (6.42)	6.06 (5.46)	7.11 (6.40)	7.22 (6.51)
10	7.85 (7.27)	5.50 (0.46)	0.00 (0.00)	8.32 (7.68)	7.88 (6.71)	7.61 (6.48)
*11	8.32 (7.28)	4.19 (0.63)	6.66 (4.91)	-	-	-
12	7.48 (6.59)	5.20 (3.97)	7.74 (7.13)	7.03 (5.15)	6.85 (6.47)	6.35 (4.96)
*13	8.10 (7.39)	6.99 (6.02)	7.81 (6.95)	7.91 (6.97)	7.30 (6.25)	7.96 (6.96)
*14	9.20 (8.83)	6.71 (4.83)	8.35 (7.32)	9.11 (8.46)	9.03 (8.23)	8.74 (8.09)
15	7.14 (5.18)	6.31 (4.22)	7.22 (5.44)	-	-	-

Notes:

*Denotes stream designated as a Coldwater Fisheries Resource (CFR), which require minimum dissolved oxygen concentrations of 6 mg/L.

**Table 5:** Differences in dissolved oxygen (DO) diel ranges between impoundment and downstream reaches and upstream references before and after dam removal, termed impoundment or downstream range effect. Range effect is the average difference between diel ranges of impacted and upstream reaches. Positive values indicate a higher diel DO range relative to upstream.

Site	Impoundment						Downstream					
	Before Removal			After Removal			Before Removal			After Removal		
	Range Effect (mg/L)	*P*	n	Range Effect (mg/L)	*P*	n	Range Effect (mg/L)	*P*	n	Range Effect (mg/L)	*P*	n
1	0.45	0.318	8	0.53	0.089	6	-0.27	0.075	8	0.49	0.052	6
2	**4.28**	0.002	14	**1.75**	0.002	12	**2.01**	0.001	14	**1.54**	0.002	12
3	0.25	0.463	24	-	-	–	-0.12	0.091	24	-	-	-
4	0.07	0.715	32	-	-	–	**-0.69**	0.001	32	-	-	-
5	0.18	0.795	8	-0.01	1.000	6	**-0.65**	0.026	8	0.01	0.916	6
6	-0.59	0.795	8	**4.99**	0.063	6	-0.77	0.078	8	3.71	0.052	6
7	-1.42	0.171	8	-	-	–	**9.05**	0.017	8	-	-	-
8	-0.11	0.795	8	-0.18	0.156	6	**-2.12**	0.017	8	-0.5	0.052	6
9	0.02	0.945	8	-0.22	0.089	6	-0.85	0.059	8	-0.2	0.078	6
10	1.94	0.094	8	**0.39**	0.002	18	-0.46	0.209	8	**0.78**	<0.001	18
11	**3.50**	<0.001	24	-	-	–	**0.47**	0.017	24	-	-	-
12	**3.19**	0.001	14	-0.04	0.486	6	**-0.35**	0.003	14	-0.01	0.916	6
13	0.30	0.463	6	1.73	0.063	6	0.09	0.438	6	0.68	0.078	6
14	**2.09**	0.009	14	**0.15**	0.047	8	**0.16**	0.021	14	**0.38**	0.026	8
15	-0.01	0.795	18	-	-	–	**-0.51**	0.001	18	-	-	-

Notes: Bold font indicates a significant difference between impoundment diel ranges (e.g., impoundment-upstream) at α = 0.05 as determined by Wilcoxon Rank-Sign tests. Column heading “n” indicates number of daily measurements included in analyses (e.g., first/last day of each logger deployment). Dash symbols (-) indicate no dam removal occurred over the course of this study.

Negative DO effects within impoundments did not translate to consistent negative effects downstream, with 4 of 15 sites exhibiting significantly reduced downstream DO compared to upstream reaches (Table E in S1 Appendix). The only site that experienced a significantly positive impoundment effect also experienced a positive downstream effect (Fig 2B). Across the 15 sites, the downstream DO effects were on average 0.51 mg/L lower, but much more variable (range = -8.20 to +3.28 mg/L) than upstream reaches (Table E in S1 Appendix). Over half of all downstream reaches (8 of 15) experienced low DO (<5 mg/L) for at least one day of the study period; of those, 4 exhibited average minimum DO < 5mg/L, and 3 of those could be considered impaired relative to upstream (Table 4). One site (site 10) experienced consistently low DO (average daily minimum: 0.00 mg/L) due to lack of water spilling over the dam. A dampened downstream diel DO range was observed at 10 of 15 sites (i.e., more stable daily DO concentrations), while 5 sites exhibited larger diel ranges compared to upstream (Table 5). Site 7—a head-of-tide dam—is notable for its extremely high diel variability downstream of the dam (avg. downstream diel range of 9.05 mg/L).

### Drivers of inter-site variation in dam effects

The variation in magnitude of impoundment effect was best explained by three highly ranked models, of which the top-ranked model (lowest AICc) was an additive model of impoundment widening (negative), percent cultivated land cover (negative), and area-normalized daily discharge with random intercept terms for site and year (Table 6). Slope estimates were such that sites with wider impoundments or more cultivated watersheds were predicted to have lower impoundment DO relative to upstream concentrations (Table 7). Two equally well supported models (i.e., ΔAICc < 2.0) contained the individual predictor variables and discharge. Additionally, there was a slight negative relationship (β = -0.17 (± 0.08 SE); df = 166.71; *p* = 0.033)) between impoundment warming (mean summer impoundment temperatures minus upstream temperatures) and the impoundment DO effect, whereby sites with warmer impoundments relative to upstream also experienced more negative DO impoundment impacts (S1 Fig).

**Table 6:** Summary of highly ranked models (ΔAICc ≤ 2.0) explaining the variation in impoundment and downstream mean dissolved oxygen (DO) effects prior to dam removal.

Pre-Removal DO Effect Models	AICc	K	ΔAICc	Marginal R^2^	Conditional R^2^
*Impoundment Effect*					
Impoundment Widening + % Cultivated Cover + Q	609.77	7	0.00	0.27	0.57
% Cultivated Cover + Q	609.85	6	0.08	0.21	0.57
Impoundment Widening + Q	611.45	6	1.68	0.12	0.55
*Downstream Effect*					
Impoundment Effect + % Wetland Cover + Q	485.92	7	0.00	0.36	0.68
Impoundment Effect + % Impervious Cover + Q	486.51	7	0.60	0.35	0.68
Impoundment Effect + Q	487.87	6	1.95	0.19	0.65

Notes: Q represents scaled and log-transformed area-normalized daily discharge, included as a fixed covariate in all models. Predictor variables were scaled to report relative and comparable effects on the response variables across all parameters. All models included random intercept terms for site and year.

**Table 7:** Standardized parameter estimates for the two top-ranked candidate models explaining variation in impoundment and downstream dissolved oxygen (DO) effects before dam removal. Estimates of fixed effects are shown with standard errors (SE), 95% confidence intervals (CI), and p-values (*P*).

DO Effect Predictors	Estimate (SE)	95% CI	*P*
*Top Impoundment Model*			
(Intercept)	-1.06 (0.33)	-1.70–-0.42	0.007
Impoundment Widening	-0.50 (0.28)	-1.06–0.06	0.104
% Cultivated Cover	-0.70 (0.32)	-1.33–-0.08	0.052
Normalized Discharge (Q)	0.02 (0.13)	-0.22–0.27	0.855
*Top Downstream Model*			
(Intercept)	0.73 (0.28)	0.14–1.32	0.018
Impoundment Effect	0.36 (0.05)	0.25–0.47	<0.001
% Wetland Cover	0.54 (0.22)	0.11–0.98	0.014
Normalized Discharge (Q)	0.17 (0.09)	-0.01–0.35	0.063

Predictor variables were scaled to report relative and comparable effects on the response variables across all parameters. All models included random intercept terms for site and year.

Variation in the downstream DO effect was best explained by a mixed effects model with impoundment effect (positive), percent wetland cover (positive), and area-normalized daily discharge with random effects for site and year (Table 6). Two other models were considered equally plausible (i.e., ΔAIC_c_ < 2.0), and these models contained watershed impervious cover in place of watershed wetland cover (also a positive relationship), and a single parameter model including pre-removal impoundment effect (Table 6). There was a positive relationship with impoundment effect such that sites with reduced impoundment DO were predicted to have lower downstream DO, both relative to upstream concentrations. There was also a positive relationship with wetland cover, whereby sites in watersheds with less wetland cover experienced greater losses of downstream DO relative to upstream (Table 7; S3 Fig).

### Stream DO responses to dam removal

**Fig 3:**
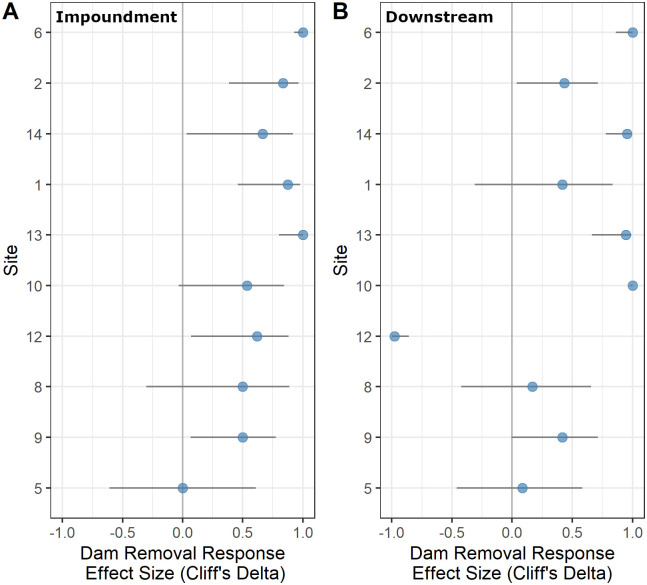
Magnitude and direction of Cliff’s delta effect size, which was used to assess the dam removal dissolved oxygen (DO) response in A) the impoundment, and B) downstream. Positive values indicate an increase in mean impoundment or downstream DO relative to upstream after dam removal. Gray bars indicate 95% confidence interval. Cliff’s delta values can be interpreted as negligible (|<0.147|), small (|<0.33|), medium (|<0.474|) and large (|0.474–1.000|) effects. Sites are ordered from lowest (bottom) to highest (top) pre-removal impoundment effect.

Across the 10 sites where dams were removed, all former impoundments experienced an increase in DO relative to their upstream reaches, resulting in impoundment DO concentrations that were more similar to, or in some cases, higher than upstream concentrations (Fig 2A). Prior to removal, 9 of 10 sites had significantly lower DO within impoundments relative to upstream reaches; following removal, all but 2 impoundments recovered to have similar DO as upstream reaches with one site (site 2) surpassing upstream concentrations (Fig 2A; Table 3). In terms of effect size, 9 of 10 impoundments experienced a large, positive DO response to dam removal (Fig 3A; Table F in S1 Appendix). Further, this recovery occurred at most sites within a year following dam removal (i.e., by the next summer sampling event after removal; S2 Fig. Dam removal improved water quality impairment at one impoundment in this study (site 2), with average minimum values increasing from 1.12 mg/L to 6.80 mg/L, above the 5 mg/L threshold. However, despite the lack of shifts in water quality impairment at most sites, the average minimum DO values across impoundments with dam removals were greatly increased from 4.90 to 6.96 mg/L (Table 4). Impoundments that were most negatively impacted by dams also experienced the largest magnitude of recovery following dam removal, with recovery ranging from 0.19 (site 5) to 5.73 mg/L (site 6; Table F in S1 Appendix). At the 3 of 10 removal sites in which impoundment diel DO ranges were significantly higher than upstream conditions, only 1 (site 12) exhibited full recovery following dam removal (e.g., became similar to upstream conditions; Table 5). Overall, there were inconsistent patterns in impoundment DO diel ranges responses after dam removal, with variability in both the magnitude and direction of response (Table 5, S4 Fig).

While impoundment DO consistently increased following dam removal, downstream responses—where DO impacts were mixed prior to removal—were similarly mixed following dam removal (Fig 2B). Most sites (8 of 10) experienced increases in Cliff’s delta after dam removal, indicating positive effects on downstream DO relative to upstream (Fig 3; Table F in S1 Appendix). Although the magnitude of responses differed across sites, generally those sites which had negative downstream DO effects prior to removal exhibited a positive response to removal (i.e., an increase in downstream DO relative to upstream). The site with the most negative downstream DO effect (site 10) had the largest positive response after removal, with the downstream effect increasing from– 8.20 to -0.38 mg/L (Fig 2B; Table E in S1 Appendix). Of the 7 sites with muted downstream diel variations (negative downstream effects) prior to dam removal, 6 experienced an increase in diel range with respect to upstream following dam removal (Table 5).

### Drivers of inter-site variation in dam removal responses

The variation in impoundment DO response to dam removal was best explained by three highly ranked models, of which the top-ranked model (lowest AICc) was an additive model of impoundment effect (negative), residence time (positive), and percent of watershed cultivated land cover with a random intercept term for site (Table 8). Sites with more negative DO effects before dam removal were predicted to have larger increases in DO relative to upstream concentrations after removal, and sites with shorter pre-removal residence times and a smaller percent of watershed wetland cover were predicted to have a smaller response to dam removal (Table 9; S5 Fig). Two equally well supported models (i.e., ΔAICc < 2.0) contained impoundment effect and residence time, and impoundment effect alone (Table 8). Variation in the downstream DO recovery relative to upstream was best explained by only one highly ranked model, which included pre-removal downstream effect (negative), pre-removal residence time (positive), and percent of watershed with wetland cover (positive), with a random effect for site (Table 8). No other model was considered equally plausible (i.e., ΔAIC_c_ > 2.0). Similar to the impoundment removal response model, there was a negative relationship with pre-removal downstream effect, such that sites with more negative downstream DO effects prior to removal had greater post-removal improvements in downstream DO relative to upstream concentrations (Table 9).

**Table 8:** Highly ranked linear models (ΔAIC ≤ 2.0) explaining the variation in impoundment and downstream dissolved oxygen (DO) responses after dam removal.

Dam Removal Response Models	AICc	K	ΔAICc	Marginal R^2^	Conditional R^2^
*Impoundment Removal Response*					
Impoundment Effect + Residence Time + % Wetland Cover	41.71	6	0.00	0.95	0.98
Impoundment Effect + Residence Time	41.41	5	0.70	0.88	0.98
Impoundment Effect	43.63	4	1.93	0.74	0.97
*Downstream Removal Response*					
Downstream Effect + Residence Time + % Wetland Cover	14.36	6	0.00	0.86	0.97

Predictor variables were scaled to report relative and comparable effects on the response variables across all parameters. All models included a random intercept term for site. Impoundment and downstream effect refer to the pre-removal difference between impoundment and upstream DO.

**Table 9:** Standardized parameter estimates for the two top-ranked candidate models explaining variation in impoundment and downstream dissolved oxygen (DO) responses to dam removal. Estimates of fixed effects are shown with standard errors (SE), 95% confidence intervals (CI), and p-values (*P*).

Dam Removal Response Predictors	Estimate (SE)	95% CI	*P*
*Top Impoundment Model*			
(Intercept)	0.39 (0.15)	0.07–0.72	0.022
Impoundment Effect	-0.89 (0.05)	-1.00–-0.77	<0.001
Residence Time	0.59 (0.14)	0.30–0.88	0.003
% Wetland Cover	0.40 (0.13)	0.13–0.67	0.019
*Top Downstream Model*			
(Intercept)	0.39 (0.12)	0.09–0.69	0.020
Downstream Effect	-0.92 (0.03)	-0.98–-0.85	<0.001
Residence Time	0.47 (0.47)	0.15–0.79	0.011
% Wetland Cover	0.52 (0.13)	0.21–0.83	0.006

Predictor variables were scaled to report relative and comparable effects on the response variables across all parameters. All models included a random intercept term for site. Impoundment and downstream effect refer to the pre-removal difference between mean reach DO and mean upstream DO.

## Discussion

### Small dams have variable effects on stream dissolved oxygen

Small dams can create impoundments that have lower DO than adjacent, free-flowing upstream sections. Here, we found that 60% of sites exhibited lower mean daily DO within the impoundment than the upstream reach, which is supported by other studies of dammed streams that report substantially lower DO concentrations within impoundments [6, 29]. The slow-moving water within impoundments facilitates the accumulation of fine sediments and organic matter [19, 47], and the subsequent decomposition of these organic sediments can result in DO consumption [6]. Most of these impoundments were found to have warmer surface waters than their upstream reaches [8], which reduces oxygen solubility. Finally, the impoundment water is typically less turbulent than free-flowing reaches, resulting in less opportunity for aeration due to atmospheric mixing [48]. These abiotic and biotic mechanisms likely work collectively to reduce DO in impoundments.

The decreased DO concentrations within 9 impoundments translated to decreases in downstream DO at only 4 dams in this study, suggesting small, surface-release dams may self-recover negative impacts to DO, likely due to the reaeration of the impoundment water as it spills over the dam. At the sites where decreased DO persisted downstream, recovery may have been constrained by minimal reaeration from turbulence at a low dam (site 1), lack of water flow to downstream (site 10) or DO losses in the impoundment which were too great to be recovered by reaeration (sites 11 & 14). Unlike higher temperatures, which have been shown to persist from small dam impoundments to up to 4 km downstream, [8], there were generally inconsistent and minimal effects of small dams on downstream DO concentrations.

Four sites—those that were noticeably more eutrophic than other sites—experienced increased diel variation in the impoundment relative to upstream reaches, supporting results observed previously in the literature [5, 6]. Increased residence times and greater exposure to solar radiation in wide impoundments may support greater abundances of phytoplankton, algae, and macrophytes [49]. Additionally, the decrease in velocity through an impounded reach can minimize the shear forces that would otherwise break up algal communities in free-flowing riverine environments, which, in turn, can lead to high algal growth within the impoundment [50]. High rates of oxygen production during daytime photosynthesis and oxygen consumption via autotrophic respiration at night result in large daily ranges in oxygen observed within impoundments, and these daily ranges may be measured as a proxy for a waterbody’s trophic state [15]. Most sites with higher impoundment diel ranges also had elevated diel ranges that persisted in the downstream reach, consistent with findings from Maxted et al. [6]. Conversely, several sites in this study exhibited muted diel DO ranges downstream. In some streams, the constant flow of water over the spillway and the constant aeration from that process may serve to dampen diel variability in downstream reaches relative to upstream reaches. While previous studies have observed reduced diel variation of both temperature and DO downstream of hydroelectric dams relative to their upstream reaches [51, 52], this study provides the first evidence of muted diel DO variation below small, surface-release dams.

### Impoundment and watershed characteristics drive inter-site variation in dam effects

Across our study sites, dams creating wide impoundments and those located within watersheds with greater percent cultivated area experienced lower impoundment DO relative to their upstream reaches. Predictors that describe impoundment shape, like impoundment widening, may be reflective of both the thermal impact and the sediment and nutrient trapping ability of the dam. Zaidel et al. [8] found wider impoundments (relative to upstream channel widths) exhibited increased temperatures that persisted downstream, likely due to increased solar radiation and limited riparian shading. Water temperature is tightly coupled with DO concentrations, with cooler waters having a greater physical capacity to hold DO [17]; thus, higher impoundment temperatures relative to upstream likely also played a role in reduced DO concentrations, which was supported by our results (S1 Fig). Additionally, in narrow, more river-like impoundments, accumulated sediments may be flushed during high flow events to a greater extent than wider impoundments [53]. Wide impoundments may therefore accumulate and retain organic matter and fine mineral sediments, which may interact with land surface runoff from cultivated areas[19]. Runoff and accumulation of excess nutrients—including nitrogen (N), phosphorus (P), and carbon (C) common in agricultural fertilizers and livestock manure—may further increase biological oxygen demand [54, 55], even at the relatively low levels of cultivated land in our study areas (0–2.6%). Although this study focuses on human-made dams, previous research has suggested that dams created by beaver (*Castor canadensis* and *Castor fiber*) may slightly decrease impoundment and downstream DO as compared to upstream [56]. However, beaver dams may not retain nutrients (i.e., N and P) to the same extent as artificial dams [56], and thus natural impoundments may not experience the same biological oxygen demand as artificial impoundments. Moreover, since most beaver dams exist on the landscape for less than a decade [57], the persistent negative impacts on DO from beaver dams are not likely to rival those caused by prevalent older (>50 years) and more impermeable artificial dams.

Downstream DO effects of small dams were best predicted by their impoundment effects, such that impoundments with very low DO relative to their upstream reaches (i.e., > 2 mg/L lower) were predicted to experience the most downstream DO losses. We observed reduced downstream DO in less than half of the sites with low impoundment DO. In these cases, it is possible that the impoundment could act as an organic matter source, leading to a downstream oxygen sag [58, 59], although we did not find consistent, corresponding increased diel ranges at these sites. It is more likely that some small surface-release dams spill oxygen-depleted impoundment waters that persist immediately downstream (where we sampled), but that have potential for recovery further downstream given the frequency of riffles.

In addition to impoundment effects, we found two other watershed predictors (wetland and impervious cover) to be important for understanding downstream DO effects. Sites with higher wetland area or impervious cover within their watersheds may have lower upstream reference DO concentrations, and thus these sites may have a reduced potential for negative impacts downstream. Of the 5 sites with the highest wetland cover (>14%), 4 had mean upstream DO concentrations less than 6.5 mg/L (Table G in S1 Appendix). Fluvial wetlands, either naturally occurring or influenced by humans (e.g., through undersized culverts), have been shown to decrease DO concentrations through increased residence time and oxygen demand from microbial processes [60, 61], which may lead to low DO at the outflow of wetland complexes. Sites in watersheds with more impervious cover showed a similar trend, potentially due to an increase in more labile organic matter from point-source wastewater inputs and higher temperatures [54, 62–64]. These results suggest these two predictors are more descriptive of a constraint on upstream reach DO, rather than explaining mechanisms driving downstream impacts.

### Most sites recover DO after dam removal

Across all dam removal sites, we observed recovery of depleted DO in former impoundment areas relative to upstream by the summer sampling season following dam removal, which generally occurred within a period of 1 year after removal. Returning an impounded stream to a lotic state increases flow rates and mixing, reduces residence time, reduces sediment and nutrient trapping, and may eventually reduce temperature as canopy cover develops; all of these factors can lead to a more natural (e.g., riverine) DO regime and increased similarity between formerly impounded and upstream reaches [23]. Additionally, new riffles can develop in previously impounded reaches, which may further increase water turbulence, and thus, DO [16, 65, 66]. In this study, there were 2 impoundments that were previously considered impaired relative to natural background conditions for DO (< 5 mg/L), and one exhibited substantial improvement (average minimum DO 1.12 to 6.80 mg/L) suggesting the potential for dam removal to improve inland water classes under State-specific surface water criteria (e.g., 314 CMR 4.05). Similar results were reported by Tuckerman and Zawiski [21], in which partial and full removal of 2 consecutive dams on the Cuyahoga River, OH, resulted in previously impaired DO improving to levels above the State’s regulatory standard.

While DO concentrations in former impoundments trended toward upstream conditions, downstream reaches showed relatively small, but variable, responses. Prior to removal, most downstream reaches did not experience consistent DO impairment, and accordingly, downstream responses may simply represent the natural variation between reaches [15]. Although most downstream reaches saw minimal dam effects, the most severe DO impairment found across all sites and reaches in this study occurred downstream at site 10, which experienced an average daily minimum of 0 mg/L and long durations of anoxia. At this site, periods of low or no flow over the dam resulted in downstream pools of stagnant water with extremely low DO. Removal of the dam returned stream flow to the primary channel, and as such, this site experienced full DO recovery (7.61 mg/L average minimum DO), which is an extreme case that underscores the importance of discharge to stream DO concentrations. With the sheer number of small, obsolete, and unmanaged dams in the U.S., similar effects could be occurring in many stream systems, with potentially dramatic consequences for water quality and biotic assemblages [67–69]. Our results suggest DO may be one of the first critical water quality parameters to recover following dam removal, setting the stage for re-colonization by more oxygen-sensitive macroinvertebrate and fish taxa.

### Pre-removal DO effects drive post-removal responses

Across dam removal sites, we determined that streams with the most negative impoundment DO effects prior to removal experienced the greatest magnitude of DO recovery following dam removal. Given that pre-removal impoundment effects were best predicted by impoundment widening and percent of watershed area cultivated, our results suggest that streams with wide impoundments located in watersheds with a higher percent of cultivated land may benefit the most from dam removal. Studies have previously documented relatively rapid geomorphic responses to dam removal, including a reduction of channel cross-sectional area, channel incision, and sediment and nutrient export from the former impoundment [19, 70, 71]. These geomorphic changes are related to reduced solar radiation, residence time, and sediment and nutrient trapping, all of which have been shown to contribute to increased DO [72]. By linking geomorphic and water quality responses, our results suggest dam removal may be used to achieve relatively rapid DO improvements, with stream DO concentrations constrained by watershed context and land cover [73, 74].

Where dams are the primary stressor, dam removals have a greater potential to improve water quality. For example, watershed size was determined to be a significant predictor of impoundment DO response to dam removal, with sites in smaller watersheds predicted to experience greater increases in impoundment DO relative to upstream. In this study, smaller watersheds generally comprised headwater streams with cooler, oxygen-rich waters [8, 75]; therefore, sites with smaller upstream watersheds likely represent greater potential for impact and subsequent recovery due to naturally higher DO concentrations within the cooler, steeper streams. Pre-removal residence time was also a significant driver of impoundment and downstream response to dam removal, such that sites that had a very short residence time prior to removal (i.e., site 5) experienced less improvements in DO. This suggests streams in which DO is minimally impacted by the presence of a dam may exhibit a smaller response following dam removal because they are already functioning at their maximum ecological potential as determined by the larger watershed context and land use.

### Implications

Within this study, negative DO impacts of small dams were limited to a subset of sites and at discrete locations in space (primarily within the impoundment); however, the impacts that do occur may have negative consequences for stream ecosystems, including the ability of organisms to survive. Most impoundments that experienced reduced surface DO also had low DO throughout the water column and near the sediment-water interface, which has direct and indirect impacts on aquatic organisms and biotic assemblages. Anoxic sediments within these impoundments can serve as hotspots for mercury methylation, the process via which mercury is converted to methylmercury [76, 77], thereby bioaccumulating up the food chain [78]. Fish with hypoxic stress experience reduced feeding and growth rates [79–81], compromised immune systems [82], and reproductive impacts [83], which indirectly result in population effects. Due to taxon-specific respiratory physiology and morphology, aquatic macroinvertebrates exhibit a high diversity of sensitivities to dissolved oxygen concentrations [84, 85], which can result in a dominance of tolerant taxa and thus, altered macroinvertebrate assemblages and associated ecological functions [55]. At the extreme, if DO levels drop below any organism’s lower tolerance limit, metabolic functions cannot be sustained and the organism will die, resulting in a loss of individuals and, potentially, loss of an entire population or assemblage [68].

Due to the negative ecological consequences of low DO and the increasing interest in aquatic restoration through dam removal, it is important for restoration practitioners to be able to understand impacts of small dams and to better predict benefits of their removal. This study found that DO impacts may be predicted using impoundment widening, cultivated area, and discharge, which are all metrics that may be determined from publicly available data. Given pre-removal impoundment effect was an important predictor of DO removal response, it is feasible for restoration practitioners to use the models presented here, along with baseline data, to predict potential DO benefits of a particular dam removal project. Further, the range of stream characteristics and geographic spread of the sites included in this study suggest that these results may be applicable to other regions in the U.S. and globally with a similar climate and moderate cultivated land cover. When collecting baseline DO for dam removal projects, our results suggest continuous monitoring for at least 2 full days during each of July, August, and September may be necessary to capture variability between months and more accurately quantify dam impacts. Due to the influence of discharge on DO impacts of small dams, it may also be useful to monitor for multiple years to capture periods of drought and high precipitation and better characterize the transient, but extreme, low DO events that may negatively affect sensitive species. Following dam removal, we observed relatively rapid improvements in DO, suggesting monitoring one summer after removal may be sufficient to determine recovery.

## Conclusions

It may be taken as a foregone conclusion that small dams negatively impact stream water quality, and that dam removal will ameliorate these negative impacts. While we found this broad statement generally holds true for DO at Massachusetts sites, we also found that there is substantial, yet predictable, variability in dam-induced water quality impacts and thus, in DO recovery following removal. Our results also suggest dam removal leads to relatively quick recovery of more natural DO regimes by the summer sampling event after removal (within 1 year), with the most impacted streams experiencing the greatest magnitudes of recovery. While this finding supports dam removal as a viable restoration action for practitioners looking to improve water quality and reduce DO impairment, these results also point to the need to set expectations for DO recovery; sites with little or no DO impact due to dams are not likely to show measurable improvements in DO following dam removal. However, since reduced DO represents only one of a suite of ecological impairments caused by small dams, consideration of other metrics—including site hydrology, sediment transport, macroinvertebrate assemblages, and fish passage—can result in a more a holistic understanding of small dam impacts and potential benefits of dam removal.

## Supporting information

S1 Fig Predicted relationship (and 95% confidence interval) between summer impoundment warming (mean daily impoundment temperature minus upstream temperature) and dissolved oxygen (DO) impoundment effect (mean daily impoundment DO minus upstream DO). Warmer impoundments tend to have lower impoundment DO, both relative to upstream (β = -0.17 ± 0.08 SE). The black line is the mean response, and the shaded polygon represents the 95% confidence interval about that mean.
(TIFF)

S2 Fig Timeline of dissolved oxygen response (DO) after dam removal. While most sites were monitored for 1 summer season after removal, site 2 was monitored for 2 summer seasons, and site 10 for 3 summer seasons after removal.
(TIFF)

S3 Fig Predictions from the best-supported model for pre-dam removal impoundment dissolved oxygen (DO) effect. The model includes: (A) percent of watershed with cultivated land cover (log-transformed), (B) impoundment widening (e.g., impoundment width:upstream width; log-transformed), and (C) area-normalized daily discharge (log-transformed). The black line is the mean response for each predictor and the shaded polygon represents the 95% confidence interval about that mean. All predictor variables were z-score standardized prior to modeling.
(TIFF)

S4 Fig Average dissolved oxygen (DO) effects (e.g., values relative to upstream) before and after dam removal in impoundment (A and C) and downstream (B and D) reaches. (TIFF)

S5 Fig Predictions from the best-supported model for post dam removal impoundment dissolved oxygen (DO) response. The model includes: (A) pre-removal impoundment DO effect, (B) pre-removal residence time (log-transformed), and (C) percent of the watershed with wetland cover. The black line is the mean response for each predictor and the shaded polygon represents the 95% confidence interval about that mean. All predictor variables were z-score standardized prior to modeling.
(TIFF)

S1 Appendix Supporting data tables. Additional data tables for *Investigating impacts of small dams and dam removal on dissolved oxygen in streams*.
(PDF)
