# Supplementary figures and images for: Investigating impacts of small dams and dam removal on dissolved oxygen in streams
Source: PLoS One. 2022 Nov 17;17(11):e0277647. doi: 10.1371/journal.pone.0277647 (PMC9671431; doi:10.1371/journal.pone.0277647)

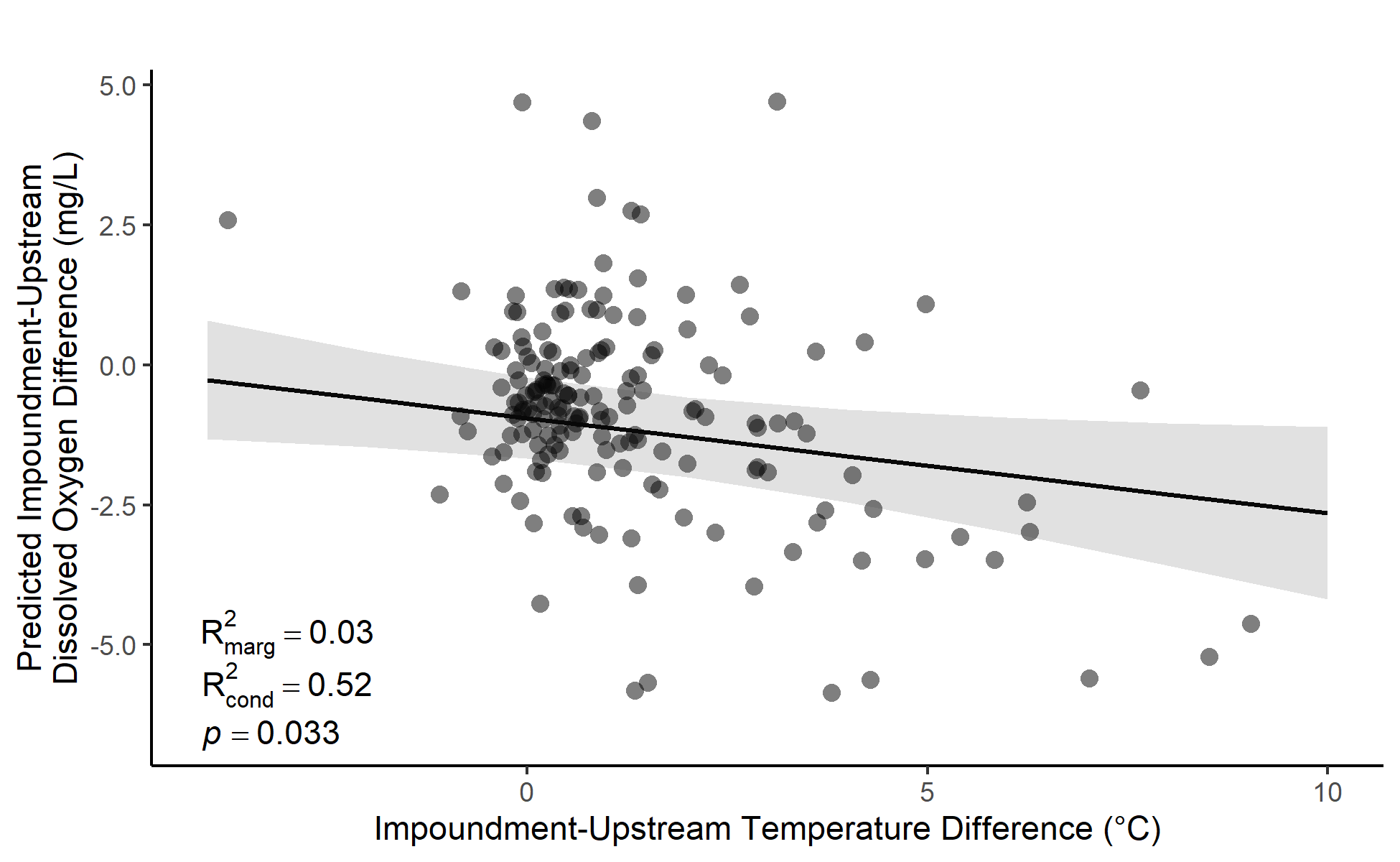

Supplement: S1 Fig — Warmer impoundments tend to have lower impoundment DO, both relative to upstream (β = -0.17 ± 0.08 SE). The black line is the mean response, and the shaded polygon represents the 95% confidence interval about that mean. (TIFF) [file pone.0277647.s001.tiff]

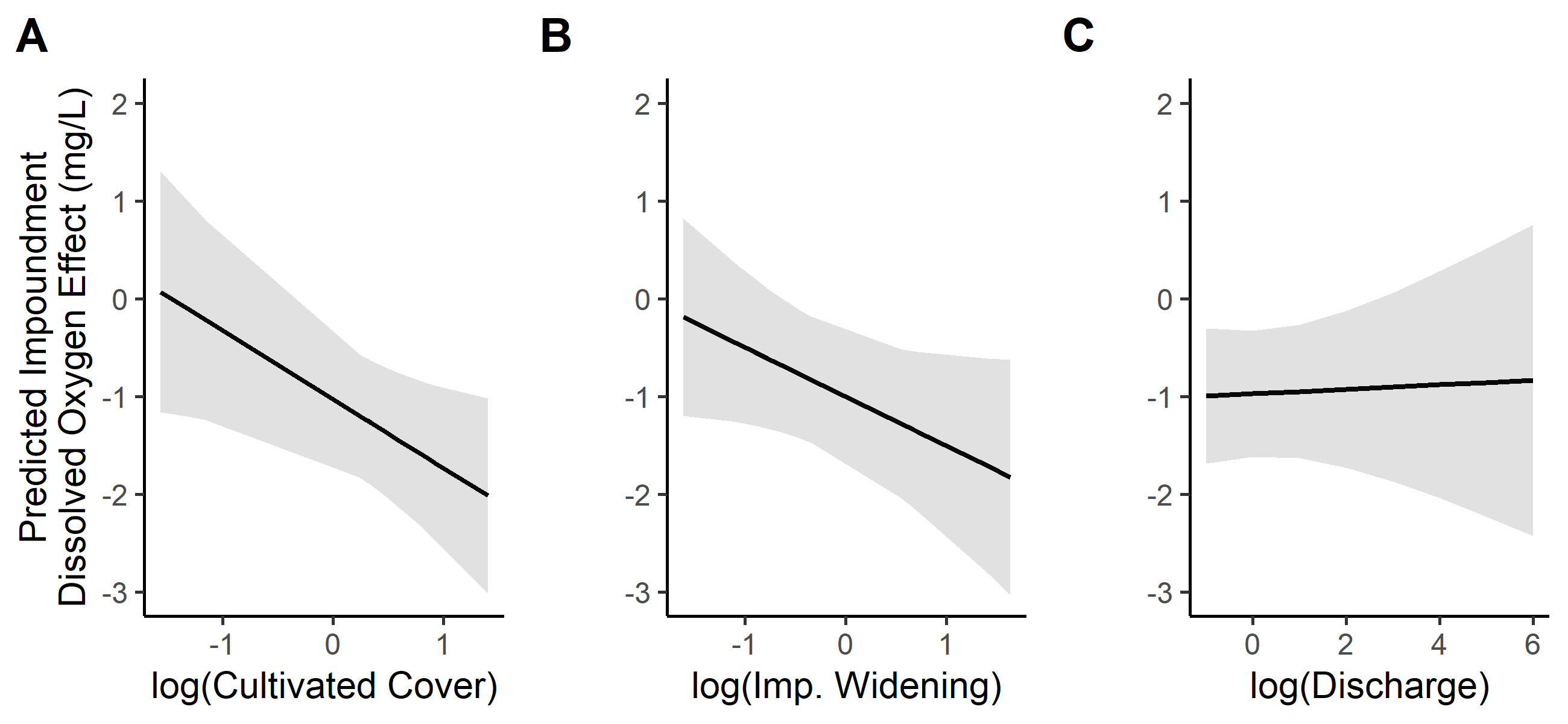

Supplement: S3 Fig — The model includes: (A) percent of watershed with cultivated land cover (log-transformed), (B) impoundment widening (e.g., impoundment width:upstream width; log-transformed), and (C) area-normalized daily discharge (log-transformed). The black line is the mean response for each predictor and the shaded polygon represents the 95% confidence interval about that mean. All predictor variables were z-score standardized prior to modeling. (TIFF) [file pone.0277647.s003.tiff]

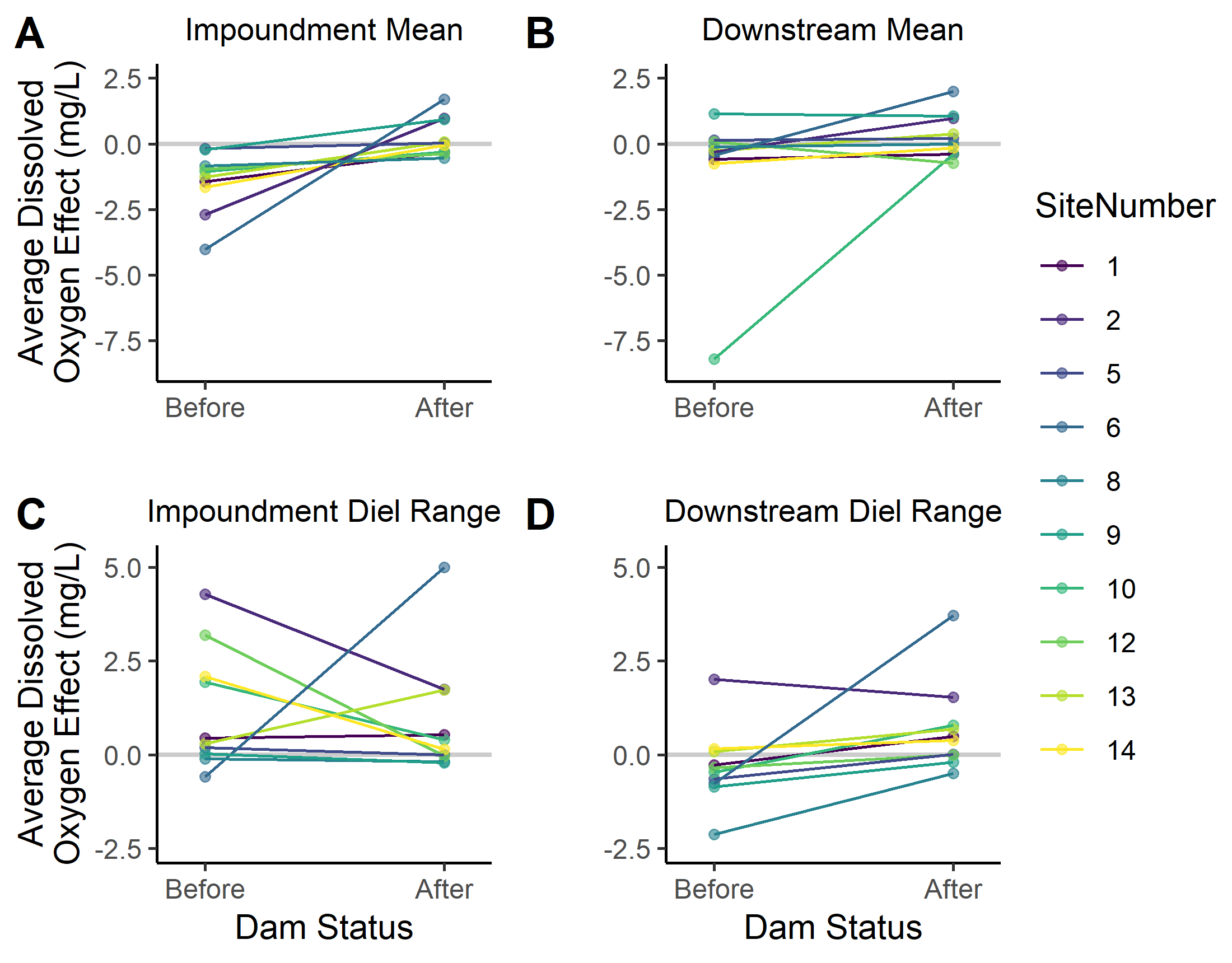

Supplement: S4 Fig — (TIFF) [file pone.0277647.s004.tiff]

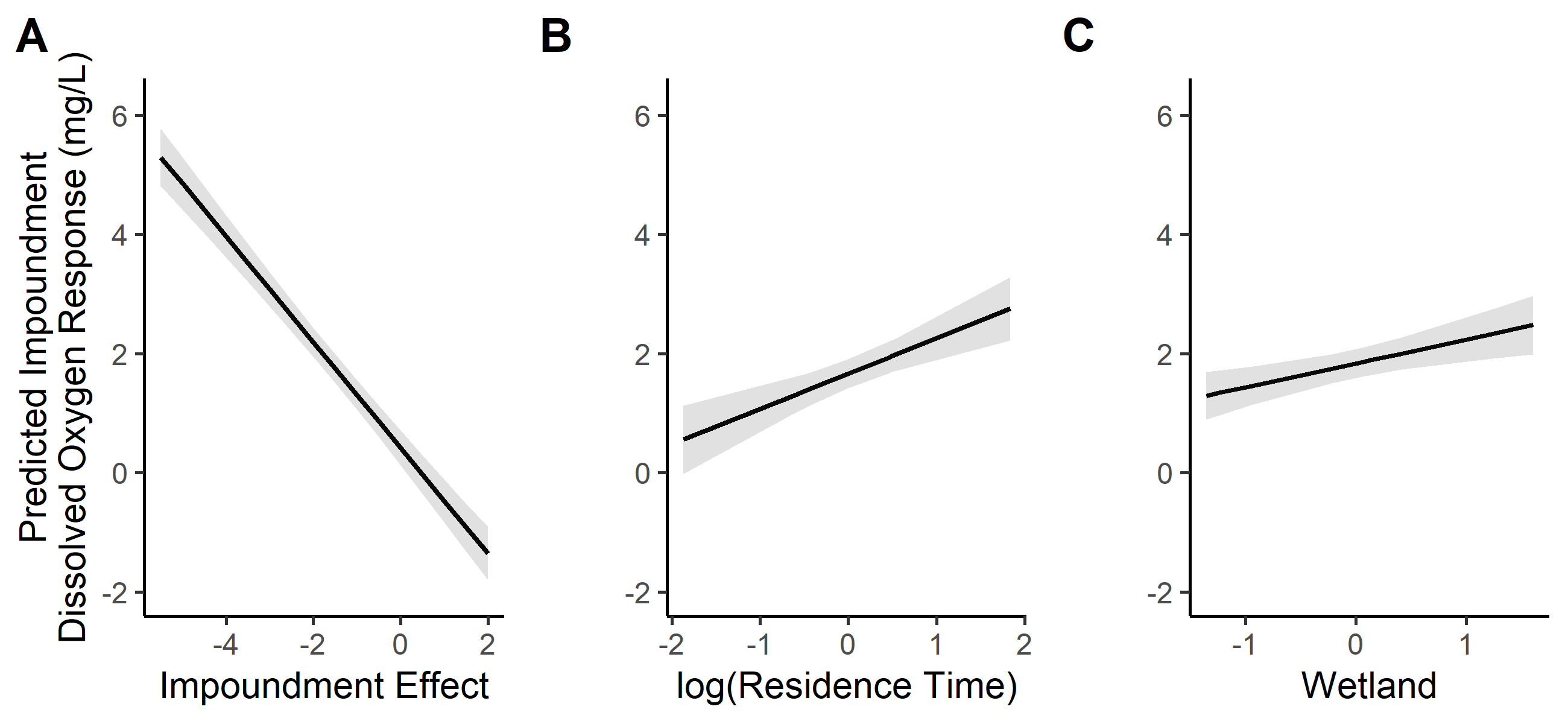

Supplement: S5 Fig — The model includes: (A) pre-removal impoundment DO effect, (B) pre-removal residence time (log-transformed), and (C) percent of the watershed with wetland cover. The black line is the mean response for each predictor and the shaded polygon represents the 95% confidence interval about that mean. All predictor variables were z-score standardized prior to modeling. (TIFF) [file pone.0277647.s005.tiff]
