# Supplementary material for: Investigating impacts of small dams and dam removal on dissolved oxygen in streams
Source: PLoS One. 2022 Nov 17;17(11):e0277647. doi: 10.1371/journal.pone.0277647 (PMC9671431; doi:10.1371/journal.pone.0277647)
Supplement: S1 Appendix — Additional data tables for Investigating impacts of small dams and dam removal on dissolved oxygen in streams. (PDF) [file pone.0277647.s006.pdf]

S1 Appendix. Supporting data tables. Additional data tables for *Investigating impacts of small dams and dam removal on dissolved oxygen in streams*.

Acknowledgements

We thank A. Ahlquist, Z. Becker, E. Chalfin, A. Grant, K. Hubbard, E. Lozier, C. Macpherson, S. Sillen, and S. Spelman for assistance in the field and laboratory. K. Ferry, A. Hackman, F. Inglefinger, B. Kelder, and N. Wildman of the Massachusetts Division of Ecological Restoration provided invaluable local knowledge that helped to establish sampling sites. We thank the Environmental Protection Agency's Regional Monitoring Network for their assistance in methods for managing and verifying data. Datasets generated during and/or analyzed during the current study are made available at U.S. Geological Survey ScienceBase at https://www.sciencebase.gov/catalog/item/6320f470d34e71c6d67aaadb. Any use of trade, firm, or product names is for descriptive purposes only and does not imply endorsement by the U.S. Government.

Table A. Number and years of ~week-long dissolved oxygen logger deployments for each site in this study. Orange shading indicates deployments occurred before dam removal, and blue shading indicates deployments occurred after dam removal. Dash symbols (-) indicate no deployments occurred over that time period. See Table 1 for site information and Figure 1 for mapped location of study sites.

Site	# Deployments Before	# Deployments After	Number of Deployments per Year						
			2015	2016	2017	2018	2019	2020	2021
1	4	3	1	3	3	-	-	-	-
2	7	6	1	3	3	3	3	-	-
3	12	-	-	-	-	3	3	3	3
4	16	-	1	3	3	3	3	3	-
5	4	3	1	3	-	3	-	-	-
6	4	3	1	3	-	3	-	-	-
7	4	-	1	3	-	-	-	-	-
8	4	3	1	3	3	-	-	-	-
9	4	3	1	3	3	-	-	-	-
10	4	9	1	3	3	3	3	-	-
11	12	-	-	-	-	3	3	3	3
12	7	3	1	3	-	3	-	3	-
13	3	3	3	3	-	-	-	-	-
14	7	4	1	3	3	1	3	-	-
15	9	-	-	-	-	-	3	3	3

Table B. Reference gage and associated watershed area used to obtain and calculate daily discharge for each site. See Table 1 for site information and Figure 1 for mapped location of study sites.

Site	Watershed Area (km^2)	USGS Gage Number	Gage Watershed Area (km^2)	Watershed Ratio
1	188.81	1100627	188.55	1.00
2	19.37	1109070	27.45	0.71
3	2.72	1199050	76.15	0.04
4	113.44	1101500	115.25	0.98
5	55.43	1105730	78.48	0.71
6	29.53	1105638	36.52	0.81
7	388.50	1102000	323.75	1.20
8	183.89	1100627	188.55	0.98
9	65.53	1103280	170.16	0.39
10	17.07	1109070	27.45	0.62
11	6.84	1096000	170.68	0.04
12	93.50	1197000	149.18	0.63
13	155.14	1096000	170.68	0.91
14	22.79	1171500	136.23	0.17
15	334.11	1173500	510.23	0.65

Notes: Daily discharge measurements were downloaded from the U.S. Geological Survey National Water Information System (NWIS; http://waterdata.usgs.gov/nwis).

Table C. Correlation coefficients between predictor variables in mixed effects modelling. Bold values indicate correlation coefficients |> 0.70|.

	Hgt	Sur	Vol	Wid	LWR	HRT	UsS	DsS	Are	For	Imp	Cul	Wet
Hgt	1												
Sur	0.16	1											
Vol	0.15	0.65	1										
Wid	-0.01	-0.42	0.02	1									
LWR	0.14	**0.86**	0.48	-0.69	1								
HRT	0.20	0.46	0.54	0.32	0.18	1							
UsS	0.32	-0.35	-0.11	-0.17	-0.28	-0.14	1						
DsS	0.20	-0.46	-0.32	0.46	-0.59	0.14	0.11	1					
Are	-0.03	0.64	0.30	**-0.83**	**0.74**	-0.16	-0.01	-0.64	1				
For	0.54	-0.01	0.33	0.43	-0.19	0.34	0.26	0.41	-0.4	1			
Imp	-0.40	0.05	-0.2	-0.45	0.24	-0.13	-0.04	-0.39	0.43	**-0.91**	1		
Cul	-0.20	0.03	0.13	0.22	-0.15	-0.23	-0.17	-0.01	0.16	0.01	-0.15	1	
Wet	-0.55	-0.11	0.01	-0.07	-0.17	0.05	-0.03	-0.19	0.26	-0.47	0.44	0.17	1

Notes: Abbreviations are: “Hgt” – Dam height, “Sur” – Impoundment Surface Area, “Vol” – Impoundment Volume, “Wid” – Impoundment Widening, “LWR” – Impoundment Length:Width Ratio, “HRT” – Impoundment Hydraulic Residence Time, “UsS” – Upstream Slope, “DsS” – Downstream Slope, “Are” – Watershed Area, “For” – Watershed Forest Cover, “Imp” – Watershed Impervious Cover, “Cul” – Watershed Cultivated Cover, and “Wet” – Watershed Wetland Cover.

Table D. Vertical profile measurements for each site before dam removal. See Table 1 for site information and Figure 1 for mapped location of study sites.

Site	# of Profiles	Avg. Profile Depth (m)	Dissolved Oxygen (mg/L)			Temperature (ºC)		
			Surface	Bottom	Difference	Surface	Bottom	Difference
1	4	1.25	6.18	4.98	1.20	22.90	22.11	0.79
2	7	0.49	5.88	4.69	1.19	21.35	20.51	0.84
3	11	1.77	7.05	5.31	1.74	15.41	10.57	4.84
4	16	1.76	4.35	1.99	2.36	26.09	20.75	5.34
5	–	–	–	–	–	–	–	–
6	2	0.40	1.66	0.74	0.92	21.87	21.49	0.38
7	5	1.36	6.42	4.68	1.74	24.69	22.88	1.81
8	4	2.25	6.44	5.31	1.13	23.34	21.81	1.53
9	4	0.75	5.58	5.10	0.48	21.49	20.96	0.53
10	–	–	–	–	–	–	–	–
11	6	0.50	6.29	5.28	1.01	20.67	19.63	1.04
12	7	1.69	6.44	6.34	0.09	21.60	21.43	0.17
13	1	1.00	8.07	8.07	0.00	18.45	17.53	0.92
14	7	3.93	7.14	0.00	7.14	20.98	13.15	7.84
15	9	2.44	6.50	4.82	1.68	20.64	19.80	0.84

Notes: Average profile depth, average surface DO and temperature, average bottom DO and temperature, and the average difference (surface – bottom) in DO between the impoundment surface and bottom at the time of the profile. Profiles not taken at sites 5 and 10 due to consistently shallow depths, as indicated by dash symbols (-).

Table E. Differences in mean daily dissolved oxygen (DO) between downstream reaches and upstream references before and after dam removal. See Table 1 for site information and Figure 1 for mapped location of study sites.

Site	Downstream DO Effect Before Removal					Downstream DO Effect After Removal				
	**Mean DO Difference (mg/L)**	***P***	**Cliff's delta *d***	**Effect Size (direction)**	**n**	**Mean DO Difference (mg/L)**	***P***	**Cliff's delta *d***	**Effect Size (direction)**	**n**
1	**-0.58**	0.023	-0.64	large (-)	8	**-0.40**	0.044	-0.33	small (-)	6
2	-0.30	0.625	-0.19	small (-)	14	**0.98**	0.002	0.79	large (+)	12
3	0.15	0.245	0.22	small (+)	24	-	-	-	-	-
4	**3.28**	<0.001	0.81	large (+)	32	-	-	-	-	-
5	0.15	0.625	0.06	negligible (+)	8	0.21	0.174	0.22	small (+)	6
6	-0.42	0.391	-0.10	negligible (-)	8	**1.99**	0.044	1.00	large (+)	6
7	-0.13	0.742	-0.41	medium (-)	8	-	-	-		-
8	-0.12	0.391	-0.06	negligible (-)	8	0.00	1.000	0.06	negligible	6
9	1.13	0.059	0.41	medium (+)	8	**1.05**	0.044	0.72	large (+)	6
10	**-8.20**	0.023	-1.00	large (-)	8	**-0.38**	<0.001	-0.49	large (-)	18
11	**-1.48**	<0.001	-0.77	large (-)	24	-	-	-	-	-
12	0.09	0.193	0.18	small (+)	14	**-0.73**	0.044	-0.56	large (-)	6
13	-0.26	0.245	-0.33	small (-)	6	**0.38**	0.044	0.39	medium (+)	6
14	**-0.75**	0.001	-0.81	large (-)	14	**-0.15**	0.044	-0.19	small (-)	8
15	-0.15	0.245	-0.15	small (-)	18	-	-	-	-	-

Notes: Bold font indicates a significant difference between impoundment diel ranges (e.g., impoundment-upstream) at α =0.05 as determined by Wilcoxon Rank-Sign tests. Column heading "n" indicates number of daily measurements included in analyses (e.g., first/last day of each logger deployment). Dash symbols (-) indicate no dam removal occurred over the course of this study. Positive values indicate an increase in mean DO relative to upstream; negative values indicate a decrease in mean DO relative to upstream. Cliff's *d* can be interpreted as negligible (|<0.147|), small (|<0.33|), medium (|<0.474|) and large (|0.474-1.000|) effects.

Table F. Differences in dissolved oxygen (DO) effects (e.g., mean impacted DO minus upstream reference DO) before and after dam removal, termed the dam removal DO response. For example, impoundment DO removal response equals the impoundment effect after dam removal minus the impoundment effect before removal. Positive values indicate that after dam removal, there was an increase in mean DO relative to upstream; negative values indicate a decrease in mean DO relative to upstream after dam removal. See Table 1 for site information and Figure 1 for mapped location of study sites.

Site	Impoundment DO Removal Response				Downstream DO Removal Response			
	**DO Response (mg/L)**	***P***	**Cliff's delta *d***	**Effect Size (direction)**	**DO Response (mg/L)**	***P***	**Cliff's delta *d***	**Effect Size (direction)**
1	**1.11**	0.016	0.88	large (+)	0.18	0.286	0.42	medium (+)
2	**3.65**	0.002	0.83	large (+)	1.28	0.107	0.43	medium (+)
5	0.19	1.000	0.00	negligible (+)	0.06	0.852	0.08	negligible (+)
6	**5.73**	0.003	1.00	large (+)	**2.41**	0.009	1.00	large (+)
8	0.32	0.238	0.50	large (+)	0.12	0.736	0.17	small (+)
9	1.16	0.172	0.50	large (+)	-0.08	0.286	0.42	medium (-)
10	0.75	0.082	0.54	large (+)	**7.82**	<0.001	1.00	large (+)
12	0.59	0.059	0.62	large (+)	**-0.82**	0.003	-0.98	large (-)
13	**1.36**	0.024	1.00	large (+)	**0.64**	0.009	0.94	large (+)
14	**1.60**	0.030	0.67	large (+)	**0.60**	0.001	0.96	large (+)

Notes: Bold font indicates a significant difference between DO effects before and after removal at α =0.05 as determined by Wilcoxon Rank-Sign tests. DO effects and n values for each group may be found in Table 4 and Table E in the S1 Appendix.

Table G. Mean dissolved oxygen (DO) concentrations, with standard deviation in parentheses, for each reach of each study site before and after dam removal. See Table 1 for site information and Figure 1 for mapped location of study sites.

Site	Mean DO Before Dam Removal (mg/L)			Mean DO After Dam Removal (mg/L)		
	Upstream	Impoundment	Downstream	Upstream	Impoundment	Downstream
1	7.89 (0.48)	6.47 (0.97)	7.31 (0.58)	8.38 (0.59)	8.06 (0.69)	7.99 (0.81)
2	5.85 (1.27)	3.33 (1.57)	5.54 (0.97)	6.83 (0.57)	7.79 (0.50)	7.81 (0.61)
3	7.84 (0.55)	7.70 (1.26)	7.98 (1.11)	-	-	-
4	3.30 (1.73)	4.26 (1.35)	6.59 (1.77)	-	-	-
5	6.94 (1.46)	6.77 (1.11)	7.09 (1.26)	6.09 (0.55)	6.18 (0.73)	6.30 (0.62)
6	5.39 (1.23)	1.37 (0.96)	4.80 (1.22)	5.68 (0.57)	7.39 (0.63)	7.67 (0.64)
7	6.91 (0.77)	6.95 (0.66)	6.78 (1.77)	-	-	-
8	8.14 (0.64)	7.30 (0.43)	8.02 (0.55)	8.28 (0.46)	7.75 (0.53)	8.28 (0.50)
9	6.79 (1.96)	6.57 (1.40)	7.93 (1.04)	6.63 (0.66)	7.56 (0.52)	7.68 (0.60)
10	8.28 (0.45)	7.15 (0.88)	0.08 (0.18)	8.63 (0.42)	8.32 (0.49)	8.25 (0.41)
11	8.80 (0.57)	5.98 (1.77)	7.34 (0.94)	-	-	-
12	7.98 (0.31)	7.01 (0.59)	8.06 (0.30)	8.02 (0.29)	7.65 (0.57)	7.29 (0.65)
13	8.38 (0.82)	7.39 (0.68)	8.12 (0.77)	8.19 (0.99)	8.28 (1.00)	8.57 (0.93)
14	9.48 (0.34)	7.88 (1.30)	8.73 (0.52)	9.42 (0.34)	9.38 (0.46)	9.27 (0.41)
15	7.70 (0.62)	6.84 (0.69)	7.55 (0.60)	-	-	-

Notes: Dash symbols (-) indicate no dam removal occurred over the course of this study.
